# Study on the Robust Filter Method of SINS/DVL Integrated Navigation Systems in a Complex Underwater Environment

**DOI:** 10.3390/s24206596

**Published:** 2024-10-13

**Authors:** Tianlong Zhu, Jian Li, Kun Duan, Shouliang Sun

**Affiliations:** 1 College of Information Science and Engineering, Hohai University, Changzhou 213001, China; 221320010025@hhu.edu.cn (T.Z.); duankun1225@163.com (K.D.); 2 China Mobile Communications Corporation Shandong Company Ltd., Jinan 250000, China; sunshouliangsh@sd.chinamobile.com

**Keywords:** strapdown inertial navigation system, Doppler Velocity Log, integrated positioning and navigation system, error correction

## Abstract

This paper proposes an improved adaptive filtering algorithm based on the Sage–Husa adaptive Kalman filtering algorithm to address the issue of measurement noise characteristics impacting the navigation accuracy in strapdown inertial navigation system (SINS)/Doppler Velocity Log (DVL) integrated navigation systems. Addressing the non-positive definite matrix problem prevalent in traditional adaptive filtering algorithms and aiming to enhance measurement noise estimation accuracy, this method incorporates upper and lower thresholds determined by a discrimination factor. In the presence of abnormal measurement data, these thresholds are utilized to adjust the covariance of the innovation, subsequently re-estimating the system’s measurement noise through a decision factor based on the innovation. Simulation and experiment results demonstrate that the proposed improved adaptive filtering algorithm outperforms the classical Kalman filter (KF) in terms of navigation accuracy and stability. Furthermore, the filtering performance surpasses that of the Sage–Husa algorithm. The simulation results in this paper show that the relative position positioning error of the improved method is reduced by 49.44% compared with the Sage–Husa filtering method.

## 1. Introduction

With the rapid advancement of ocean exploration technology and the expanding demands of ocean development, Autonomous Unmanned Vehicles (AUVs) have emerged as the primary focus of future underwater vehicle development and research. Consequently, underwater vehicle navigation systems are facing increasingly stringent requirements [1,2]. However, traditional radio navigation technology and space-based navigation technology face significant limitations in underwater navigation due to the complexity of the underwater environment and the high attenuation of electromagnetic wave propagation in water [3]. SINS serves as an autonomous navigation estimation system that offers accurate positioning and navigation information in environments with minimal external signal interference [4]. By utilizing inertial components such as gyroscopes and accelerometers, SINS computes real-time navigation parameters for underwater carriers through strapdown solutions [5]. Nevertheless, the accumulation of inertial measurement errors during navigation computations gradually degrades the system’s navigation performance, making pure inertial navigation inadequate for long underwater journeys and achieving long-distance navigation accuracy [6]. Recognizing the inherent limitations and operational constraints of individual sensors, the task of measuring underwater vehicle navigation information necessitates the integration of multiple pieces of navigation equipment. DVL, an underwater speed measurement instrument leveraging the underwater acoustic Doppler effect, provides stable and accurate speed measurement results by enabling both convection and bottom speed measurements [7,8]. The fusion of various navigation information enhances the reliability and accuracy of the vehicle’s navigation results.

Currently, the SINS/DVL combination scheme predominantly serves as an underwater integrated positioning and navigation solution. Acting as an external auxiliary speed measurement instrument, DVL furnishes real-time velocity measurement data, which mitigates the accumulation of SINS time integral errors and enhances navigation equipment performance through the estimation of combined SINS parameters [9]. However, in complex underwater environments, changes in external noise characteristics can lead to abnormal DVL data, significantly impacting navigation accuracy. Instances of new invalid outliers in DVL speed measurements leave only the Inertial Navigation System (INS) to function independently within the integrated navigation system, resulting in divergent navigation accuracy.

To address the challenges encountered in SINS/DVL integrated navigation systems, adaptive filtering stands out as the most effective solution currently available. Among the various adaptive filtering algorithms, Sage–Husa adaptive filtering is widely employed, as it attenuates the impact of real-time changes on navigation accuracy by adjusting the system’s noise characteristics [10]. However, in traditional adaptive filtering algorithms, the choice of the fading factor significantly affects the system’s parameter estimation performance. Pre-setting the fading factor in previous algorithms often resulted in inaccurate estimations, particularly in dynamically changing underwater environments [11]. Furthermore, conventional measurement noise estimation algorithms may encounter issues such as a negative positive definite matrix when the innovation is minimal, ultimately leading to navigation accuracy divergence [12]. In order to overcome these challenges, Wang D proposed in [13] to identify each element of the diagonal of the measurement noise matrix to overcome the problem of negative positive definite matrix. In [14], it is proposed to use innovation to distinguish abnormal measurement data. However, the discriminant factor is not used to dynamically adjust the measurement noise.

This paper proposes a novel approach that integrates innovation and establishes upper and lower limits for decision factors, making full use of discriminant factors to estimated measurement noise. By devising a measurement noise estimation method based on these decision factors, a new improved adaptive filtering algorithm is introduced, enhancing the system’s robustness and adaptability.

This novel methodology effectively mitigates the impact of real-time changes on navigation accuracy by refining the estimation process. By dynamically adjusting the fading factor and employing a decision-based approach for measurement noise estimation, the proposed algorithm demonstrates enhanced resilience in complex underwater environments. Overall, this advancement significantly improves the performance of SINS/DVL integrated navigation systems, ensuring reliable and accurate navigation outcomes.

## 2. SINS/DVL Integrated Navigation System Model

### 2.1. Integrated Navigation Filtering Algorithm

Currently, the Kalman filter is a commonly employed method in SINS/DVL integrated navigation systems to integrate data from various navigation sensors. Despite its unsuitability for nonlinear systems, this does not preclude its applicability in integrated navigation scenarios. However, if the state variables of the system are directly chosen as navigation parameters, or if the system and measurement noises are not Gaussian white noise, the standard Kalman filter becomes impractical. In such cases, selecting an appropriate filtering algorithm according to the specific circumstances is necessary.

Two methods exist for selecting state variables in the SINS/DVL navigation system: the direct method and the indirect method. The direct method utilizes the output parameters of the navigation subsystem directly as the state variables of the combined system, which are then fed into the filter for computation. However, since the system equation describing the state is nonlinear, the direct method is only suitable for linear systems with Kalman filtering. For nonlinear systems, alternative filtering methods must be employed, thereby increasing the complexity of practical applications [15].

Conversely, the indirect method employs the differences in navigation subsystem parameters as state variables and feeds them into the filter for navigation estimation. As the mathematical model with differences as variables is linear, the standard Kalman filter is highly suitable in this scenario, offering practical engineering implementation ease. Therefore, when both the system and measurement noises adhere to Gaussian white noise characteristics, and navigation parameter errors are selected as the system’s state variables, the coupling term between them can be neglected due to their small magnitude. Consequently, the integrated navigation system simplifies into a linear system, enabling the use of the standard Kalman filter.

In practice, the standard Kalman filter can adequately handle most integrated navigation applications, offering low computational complexity and superior real-time performance, making it a preferred choice for many scenarios [14,16].

**Figure 1:**
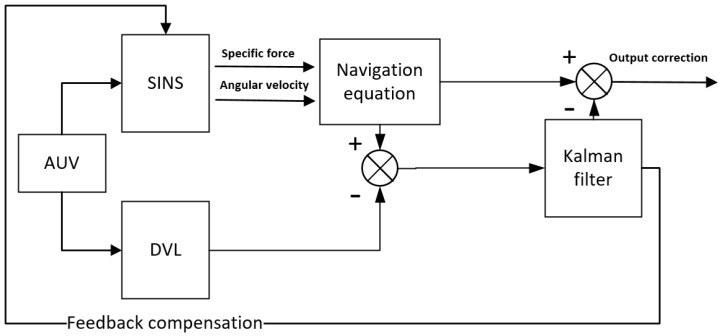
Hybrid calibration diagram of integrated navigation system.

As illustrated in Figure 1, an SINS/DVL integrated navigation hybrid correction model is implemented utilizing output correction and feedback correction mechanisms within the integrated navigation system. During the initial oscillation stage of filtering, output correction is applied. As the filter gradually stabilizes and the estimation error falls within the acceptable range, feedback correction is initiated [17]. This approach allows for both the high-frequency updating of strapdown inertial navigation system parameters and the correction of estimation errors through feedback mechanisms, thereby ensuring the utmost accuracy and stability of navigation.

### 2.2. Mathematical Model SINS Error

The error model of the SINS/DVL integrated navigation system encompasses two primary components: the SINS error model and the DVL error model. The inertial sensor within the strapdown inertial navigation system typically comprises three gyroscopes and three accelerometers [18,19]. However, owing to machining and assembly inaccuracies, there exists an installation error angle between the gyroscope axis and the axis of the ideal carrier coordinate system.

#### 2.2.1. Error Model of Inertial Device

(a) Gyroscope error model

The scale factor error of a gyroscope can be effectively approximated by a constant value, which is typically represented as a random constant error introduced in practical applications, denoted as

(1) $$\delta K_{G_{i}}=0\;(i=x,y,z)$$

The angular motion of the carrier is measured by the gyroscope, and the accuracy of the gyroscope directly impacts the measurement accuracy of the vehicle’s attitude parameters. Gyroscopic drift consists of deterministic drift and random drift. Deterministic drift is relatively straightforward to compensate for post-calibration, whereas real-time compensation for random drift is typically achieved through filtering technology. Its random model can be represented as follows:

(2) $$\varepsilon_{i}=\varepsilon_{bi}+\varepsilon_{ri}+\varepsilon_{gi}$$

where, $\varepsilon_{bi(i=x,y,z)}$ is a random constant; $\varepsilon_{ri(i=x,y,z)}$ is slow change drift and $\varepsilon_{gi}$ is fast change drift, both of which are white noise.

(3) $$\begin{array}{l} {\overset{-}{\varepsilon }}_{ri}=-\frac{1}{\tau_{ri}}\varepsilon_{ri}+w_{ri}\\ {\overset{-}{\varepsilon }}_{bi}=0 \end{array}$$

where $\tau_{ri}$ is the correlation time, and when the correlation time is large, its reciprocal can be regarded as a zero value; $\varepsilon_{ri}$ is approximated as a random constant, its derivative is zero; and $w_{ri}$ is the sum of the white noise.

The error model of the gyroscope can ultimately be simplified to the sum of random constant errors and white noise, expressed as

(4) $$\begin{array}{l} \varepsilon_{i}=\varepsilon_{bi}+w_{ri}\\ {\overset{-}{\varepsilon }}_{bi}=0 \end{array}$$

(b) Error model of accelerometer

Compared to the gyroscope, the accelerometer typically exhibits a higher measurement accuracy. Generally, only the zero-bias $\nabla_{i}$ of the accelerometer is taken into account, and its error model can also be represented as the “random constant + white noise” model:

(5) $$\left\{\begin{array}{l} \nabla_{i}=\nabla_{bi}+w_{gi}\\ {\overset{-}{\nabla }}_{bi}=0 \end{array}\right.$$

where $\nabla_{bi}$ is a random constant and $w_{gi}$ is white noise.

#### 2.2.2. Attitude Error Equation

It is assumed that the ideal attitude matrix from the navigation coordinate system (*n* system) to the carrier coordinate system (*b* system) is $C_{b}^{n}$, and the attitude matrix calculated by the computer simulation platform of the strapdown inertial navigation system is ${\tilde{C}}_{b}^{n}$, and the deviation between the two is called the attitude error.

The attitude differential equation of the strapdown inertial navigation system is as follows:

(6) $$\begin{array}{ll} {\dot{C}}_{b}^{n} & =C_{b}^{n}\left(\omega_{nb}^{b}\times \right)\\ & =C_{b}^{n}\left[\begin{array}{ccc} 0 & -\omega_{nby}^{b} & \omega_{nby}^{b}\\ \omega_{nbz}^{b} & 0 & -\omega_{nbx}^{b}\\ -\omega_{nby}^{b} & \omega_{nbx}^{b} & 0 \end{array}\right] \end{array}$$

However, under the influence of error sources in application, the actual attitude error equation of the system is as follows:

(7) $${\dot{\tilde{C}}}_{b}^{n}={\tilde{C}}_{b}^{n}\left({\tilde{\omega }}_{nb}^{b}\times \right)$$

In the above formula,

(8) $$\begin{array}{l} {\tilde{\omega }}_{nb}^{b}={\tilde{\omega }}_{ib}^{b}-{\tilde{C}}_{n}^{b}{\tilde{\omega }}_{in}^{n}=\left(\omega_{ib}^{b}+\delta \omega_{ib}^{b}\right)-{\tilde{C}}_{n}^{b}\left(\omega_{in}^{n}+\delta \omega_{in}^{n}\right)\\ \left({\tilde{\omega }}_{nb}^{b}\times \right)=\left[\begin{array}{ccc} 0 & -{\tilde{\omega }}_{nbz}^{b} & {\tilde{\omega }}_{nby}^{b}\\ {\tilde{\omega }}_{nbz}^{b} & 0 & -{\tilde{\omega }}_{nbx}^{b}\\ -{\tilde{\omega }}_{nby}^{b} & {\tilde{\omega }}_{nbx}^{b} & 0 \end{array}\right] \end{array}$$

where $\delta \omega_{ib}^{b}$ is the error of gyro measurement, $\omega_{in}^{n}$ is the projection of the angular velocity of the inertial coordinate system relative to the navigation coordinate system in the navigation coordinate system, $\delta \omega_{in}^{n}$ is the calculation error of $\omega_{in}^{n}$, and $\delta \omega_{in}^{n}=\delta \omega_{ie}^{n}+\delta \omega_{en}^{n}$.

The calculation error of the strapdown matrix can be defined as below:

(9) $$\Delta C={\tilde{C}}_{b}^{n}-C_{b}^{n}={\tilde{C}}_{b}^{n}-C_{b}^{n}{\tilde{C}}_{b}^{n}=\left(I-{\tilde{C}}_{b}^{n}\right){\tilde{C}}_{b}^{n}$$

We differentiate ${\tilde{C}}_{b}^{n}-C_{b}^{n}$ in Equation (9) and substitute (6) to obtain the following:

(10) $$\Delta \dot{C}={\tilde{\dot{C}}}_{b}^{n}-{\dot{C}}_{b}^{n}=C_{b}^{n}\left({\tilde{\omega }}_{nb}^{b}\times \right)-C_{b}^{n}\left(\omega_{nb}^{b}\times \right)$$

Similarly, by differentiating $\left(I-{\tilde{C}}_{b}^{n}\right){\tilde{C}}_{b}^{n}$, we obtain the following:

(11) $$\Delta \dot{C}=-{\dot{\tilde{C}}}_{b}^{n}C_{b}^{n}+\left(I-{\tilde{C}}_{b}^{n}\right){\dot{\tilde{C}}}_{b}^{n}=-{\dot{\tilde{C}}}_{b}^{n}C_{b}^{n}+\left(I-{\tilde{C}}_{b}^{n}\right){\tilde{C}}_{b}^{n}\left({\tilde{\omega }}_{nb}^{b}\times \right)$$

The right sides of Equations (10) and (11) are equal to each other and are substituted into the actual attitude Equation (7). We multiply both sides by ${\tilde{C}}_{n}^{b}$ on the left and ${\tilde{C}}_{b}^{n}$ on the right at the same time to obtain:

(12) $$\left({\tilde{\omega }}_{nb}^{b}\times \right)+{\tilde{C}}_{b}^{n}\left({\tilde{\omega }}_{nb}^{b}\times \right){\tilde{C}}_{n}^{b}-{\tilde{C}}_{b}^{n}\left(\omega_{nb}^{b}\times \right){\tilde{C}}_{n}^{b}=0$$

By leveraging the relationship between antisymmetric matrices and vectors, along with the principle of antisymmetric matrix similarity transformation, Formula (10) can be incorporated and rearranged as follows:

(13) $${\tilde{\omega }}_{nb}^{b}=\left(I-{\tilde{C}}_{n}^{b}\right){\tilde{\omega }}_{in}^{n}+{\tilde{C}}_{n}^{b}\delta \omega_{in}^{n}-{\tilde{C}}_{b}^{n}\delta \omega_{ib}^{b}$$

Finally, by substituting the above formula into the differential equation of the misalignment angle $\dot{\Phi }=C_{\omega }^{-1}{\tilde{\omega }}_{nb}^{b}$, we obtain the following:

(14) $$\dot{\Phi }=C_{\omega }^{-1}\left[\left(I-{\tilde{C}}_{n}^{b}\right){\tilde{\omega }}_{in}^{n}+{\tilde{C}}_{n}^{b}\delta \omega_{in}^{n}-{\tilde{C}}_{b}^{n}\delta \omega_{ib}^{b}\right]$$

Equation (14) is termed as the strapdown inertial navigation attitude error equation, which quantifies the degree of attitude angle misalignment between the calculated navigation system ($n^{\prime }$ system) and the ideal navigation system (n system).

#### 2.2.3. Velocity Error Equation

The speed error refers to the disparity between the calculated speed and the ideal speed within the strapdown inertial navigation system. In practical applications, accounting for the influence of errors, the actual velocity error equation of the system is expressed as follows:

(15) $${\dot{\tilde{V}}}^{n}=C_{b}^{n}{\tilde{f}}^{b}-\left(2{\tilde{\omega }}_{ie}^{n}+{\tilde{\omega }}_{en}^{n}\right)\times {\tilde{V}}^{n}+{\tilde{g}}^{n}$$

where ${\tilde{V}}^{n}=V^{n}+\delta V^{n}$, ${\tilde{f}}^{b}=f^{b}+\delta f^{b}$, ${\tilde{\omega }}_{ie}^{n}=\omega_{ie}^{n}+\delta \omega_{ie}^{n}$, ${\tilde{\omega }}_{en}^{n}=\omega_{en}^{n}+\delta \omega_{en}^{n}$, ${\tilde{g}}^{n}=g^{n}+\delta g^{n}$, $\delta f^{b}$ is the measurement error of the accelerometer, and the difference between (15) and the specific force equation for strapdown inertial navigation is sorted out to obtain the velocity error equation:

(16) $$\begin{array}{ll} \delta {\dot{v}}^{n} & ={\dot{\tilde{V}}}^{n}-{\dot{V}}^{n}\\ & =\left[I-{\left(C_{n}^{n^{\prime }}\right)}^{T}\right]C_{b}^{n^{\prime }}{\tilde{f}}^{b}+{\left(C_{n}^{n^{\prime }}\right)}^{T}C_{b}^{n^{\prime }}\delta f^{b}-\left(2\delta \omega_{te}^{n}+\delta \omega_{cn}^{\prime \prime }\right)\times {\tilde{V}}^{n}-\\ & \left(2{\tilde{\omega }}_{ie}^{n}+{\tilde{\omega }}_{cn}^{n}\right)\times \delta V^{n}+\left(2\delta \omega_{ie}^{n}+\delta \omega_{en}^{n}\right)\times \delta V^{n}+\delta g^{n} \end{array}$$

In the above calculation, the parameter error can be expressed as

(17) $$\delta \omega_{te}^{n}={\tilde{\omega }}_{te}^{n}-\omega_{tk}^{n}=\left[\begin{array}{c} 0\\ \omega_{ic}[\cos\tilde{L}-\cos(\tilde{L}-\delta L)]\\ \omega_{ie}[\sin\tilde{L}-\sin(\tilde{L}-\delta L)] \end{array}\right]$$

(18) $$\delta \omega_{en}^{n}={\tilde{\omega }}_{en}^{n}-\omega_{en}^{n}=\left[\begin{array}{c} -\delta \dot{L}\\ \dot{\tilde{\lambda }}\cos\tilde{L}-(\dot{\tilde{\lambda }}-\delta \dot{\lambda })\cos(\tilde{L}-\delta L)\\ \dot{\tilde{\lambda }}\sin\tilde{L}-(\dot{\tilde{\lambda }}-\delta \dot{\lambda })\sin(\tilde{L}-\delta L) \end{array}\right]$$

where L is latitude and λ is longitude.

#### 2.2.4. Selection of Correction Method

The deviation of the differential equation of strapdown inertial navigation position (latitude, longitude, and altitude) is computed, and the neutralization of the equation is considered quantitatively. This process yields the position error equation, which is expressed as follows:

(19) $$\left\{\begin{array}{l} \delta \dot{L}=\dot{\tilde{L}}-\dot{L}=\frac{{\tilde{V}}_{N}^{n}}{{\tilde{R}}_{M}}-\frac{{\tilde{V}}_{N}^{n}-\delta V_{N}^{\prime \prime }}{\left({\tilde{R}}_{M}-\delta R_{M}\right)}\\ \delta \dot{\lambda }=\dot{\tilde{\lambda }}-\dot{\lambda }=\frac{{\tilde{V}}_{c}^{n}\sec\tilde{L}}{{\tilde{R}}_{N}}-\frac{\left({\tilde{V}}_{E}^{n}-\delta V_{E}^{n}\right)\sec(\tilde{L}-\delta L)}{\left({\tilde{R}}_{N}-\delta R_{N}\right)}\\ \delta \overset{\cdot }{h}=\delta V_{U} \end{array}\right.$$

Among them, δL, δλ, and δh are latitude errors, longitude errors, and height errors, while $V_{E}$, $V_{N}$, and $V_{U}$ and $\delta V_{E}$, $\delta V_{N}$, and $\delta V_{U}$ are speed and speed errors in three directions, respectively.

### 2.3. Mathematical Model of DVL Error

The Doppler Velocity Log (DVL) serves as an auxiliary navigation sensor within the SINS/DVL integrated positioning and navigation system. The accuracy of speed measurement by the DVL significantly influences the overall performance of the integrated positioning and navigation system [20]. Hence, it is imperative to analyze the speed measurement error of the DVL and establish its speed measurement error model. Below is the DVL error model derived from its working principle and velocity measurement formula.

**Figure 2:**
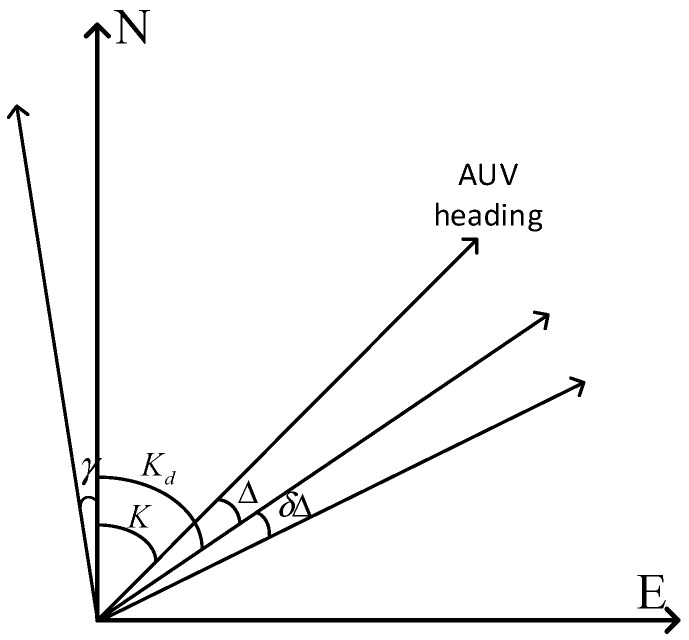
DVL error schematic diagram.

The measurement error of DVL mainly includes three parts: base velocity offset error $\delta V_{d}$, calibration coefficient error δc, and deflection angle error δΔ. The principle diagram of the velocity error of the Doppler tachometer is shown in Figure 2, where K is the true course of the AUV; $K_{d}$ is the actual track of the AUV affected by the current. Δ is the yaw angle, the so-called yaw angle is the angle between the course line and the track line of the vehicle; γ stands for the azimuth misalignment angle; and δΔ indicates deviation from the angle error.

Suppose that the velocity measured by the Doppler log against the bottom is $V_{d}$, its expression is

(20) $$V_{d}=(1+\delta c)(v_{d}+\delta V_{d})$$

where $v_{d}$ is the true velocity of motion against the base. The east and north components of $V_{d}$ on the strapdown inertial navigation platform are

(21) $$\left\{\begin{array}{l} V^{dE}=(1+\delta c)\left(V_{d}+\delta V_{d}\right)\sin\left(K_{d}+\gamma +\delta \Delta \right)\\ V_{dN}=(1+\delta c)\left(V_{d}+\delta V_{d}\right)\cos\left(K_{d}+\gamma +\delta \Delta \right) \end{array}\right.$$

Since γ and δΔ are both minimal quantities, expanding the formula gives

(22) $$\left\{\begin{array}{l} V_{dE}\approx V_{d}\sin K_{d}+V_{d}(\gamma +\delta \Delta )\cos K_{d}+V_{d}\delta c\sin K_{d}+\delta V_{d}\sin K_{d}\\ V_{dN}\approx V_{d}\cos K_{d}-V_{d}(\gamma +\delta \Delta )\sin K_{d}+V_{d}\delta c\cos K_{d}+\delta V_{d}\cos K_{d} \end{array}\right.$$

(23) $$\left\{\begin{array}{l} \begin{array}{l} V_{d}\sin K_{d}=V_{E}\\ V_{d}\cos K_{d}=V_{N} \end{array}\\ \begin{array}{l} \delta V_{d}\sin K_{d}=\delta V_{dE}\\ \delta V_{d}\cos K_{d}=\delta V_{dN} \end{array} \end{array}\right.$$

where the velocity offset error $\delta V_{d}$ and δΔ deflection angle error are both first-order Markov processes, and the calibration coefficient error δc is a random constant; then, the velocity measurement error model of the four-beam Jenners configured DVL is as follows:

(24) $$\left\{\begin{array}{l} \delta {\dot{V}}_{d}=-\beta_{d}\delta V_{d}+w_{d}\\ \delta \dot{\Delta }=-\beta_{\Delta }\delta \Delta +w_{\Delta }\\ \delta \dot{c}=0 \end{array}\right.$$

where $w_{d}$ and $w_{\Delta }$ are the white noise of the excitation Markov process, and $\beta_{d}$ and $\beta_{\Delta }$ are the reciprocal of the correlation time of the velocity deviation error and the bias angle error.

### 2.4. Mathematical Model of SINS/DVL Integrated Navigation System

Assuming that the state updating process of a physical system follows a discrete-time random process, we can define the state space model of the random system as follows:

(25) $$\left\{\begin{array}{l} {Χ}_{k}=\Phi_{k/k-1}{Χ}_{k-1}+\Gamma_{k-1}W_{k-1}\\ {Ζ}_{k}=H_{k}{Χ}_{k}+V_{k} \end{array}\right.$$

where ${Χ}_{k}$ is the state matrix; $Z_{k}$ is the measurement matrix; $\Phi_{k/k-1}$, $\Gamma_{k-1}$, and $H_{k}$ are the given system structure parameters, which are the state one-step transition matrix, the system noise distribution matrix, and the measurement matrix of the system from K-1 time to K time, respectively. $W_{k-1}$ and $V_{k}$ are system noise vectors and measurement noise vectors, respectively.

Assumptions:

(1) Both system noise $W_{k(k\geq 0)}$ and measurement noise $V_{k(k\geq 0)}$ are Gaussian white noise sequences or zero-mean white noise sequences;
(2) $W_{k(k\geq 0)}$ and $V_{k(k\geq 0)}$ are not correlated;
(3) The statistical characteristics such as the mean and variance of the initial ${Χ}_{0}$ of the system are known;
(4) Both $W_{k(k\geq 0)}$ and $V_{k(k\geq 0)}$ are independent of the initial state ${Χ}_{0}$.

It is satisfied that

(26) $$\left\{\begin{array}{l} E\left[W_{k}\right]=0,\;E\left[W_{k}W_{j}^{T}\right]=Q_{k}\delta_{kj}\\ E\left[V_{k}\right]=0,\;E\left[V_{k}V_{j}^{T}\right]=R_{k}\delta_{kj}\\ E\left[W_{k}V_{j}^{T}\right]=0 \end{array}\right.$$

where $Q_{k}$ is a non-negative definite system noise sequence variance matrix; $R_{k}$ is the variance matrix of the measured noise sequence, which requires a positive definite matrix. $\delta_{kj}$ represents the Kronecker function, which is defined as follows:

(27) $$\delta_{kj}=\left\{\begin{array}{ll} 0 & k\neq j\\ 1 & k=j \end{array}\right.$$

According to the above analysis, the system state variance model can be expressed as follows:

(28) $$\dot{X}=F_{t}X+G\omega$$

where X and ω are the system state vector and noise vector, respectively, ω is assumed to be a Gaussian white noise sequence with the mean of 0, and $F_{t}$ and G are the system state transition matrix and the system noise matrix, respectively.

According to the linear error model of the strapdown inertial navigation system, the form of the state matrix $F_{t}$ can be obtained.

We select the following error parameters to construct the state variable vector for the system state variable:

(29) $$X={\left[\phi_{x}\;\;\phi_{y}\;\;\phi_{z}\;\;\delta V_{E}\;\;\delta V_{N}\;\;\delta V_{U}\;\;\delta L\;\;\delta \lambda \;\;\delta h\;\;\nabla_{x}\;\;\nabla_{y}\;\;\nabla_{z}\;\;\varepsilon_{x}\;\;\varepsilon_{y}\;\;\varepsilon_{z}\right]}^{T}$$

where $\phi_{x}$ $\;\phi_{y}$ $\phi_{z}$ represent the attitude error, $\delta V_{E}$ $\delta V_{N}$ $\delta V_{U}$ represent east, north, and sky velocity error, respectively, δL δλ δh represent the longitude, latitude, and altitude position error, $\nabla_{x}$ $\nabla_{y}$ $\nabla_{z}$ represent the constant zero deviation of the accelerometer, and $\varepsilon_{x}$ $\varepsilon_{y}$ $\varepsilon_{z}$ represent the constant zero deviation of the gyroscope.

The measurement equation of the system is expressed as follows:

(30) Z=HX+V

Z is the measurement direction finding quantity, H is the measurement matrix, and V is the measurement noise vector. The measurement vector Z consists of the measurement speed difference between the output of INS and DVL.

(31) $$\;\;\;\;Z={\hat{V}}_{INS}^{b}-{\hat{V}}_{DVL}^{b}\approx -C_{n}^{b}\left(\phi \times \right)V^{n}+\delta V^{b}\;$$

Among them, the speed value of the INS output *b* system and the speed measurement value of DVL output are, respectively, shown in the table. The conversion of the INS output speed from the *n* system to the carrier coordinate system can be completed by the following formula:

(32) $${\hat{V}}_{INS}^{b}=C_{n}^{b}{\hat{V}}_{INS}^{n}=C_{n}^{b}\left(I-\phi \times \right)\left(V_{INS}^{n}+\delta V_{INS}^{n}\right)$$

(33) $${\hat{V}}_{DVL}^{b}=V_{DVL}^{b}+\delta b_{DVL}^{b}$$

The measurement matrix can be further derived as follows:

(34) $$H=\left[\begin{array}{ccc} H_{1} & I_{3\times 3} & 0_{3\times 9} \end{array}\right]$$

where

(35) $$H_{1}=\left[\begin{array}{ccc} C_{13}V_{DVLy}-C_{12}V_{DVLz} & C_{11}V_{DVLz}-C_{13}V_{DVLx} & C_{12}V_{DVLx}-C_{11}V_{DVLy}\\ C_{23}V_{DVLy}-C_{22}V_{DVLz} & C_{21}V_{DVLz}-C_{23}V_{DVLx} & C_{22}V_{DVLx}-C_{21}V_{DVLy}\\ C_{33}V_{DVLy}-C_{32}V_{DVLz} & C_{31}V_{DVLz}-C_{33}V_{DVLx} & C_{32}V_{DVLx}-C_{31}V_{DVLy} \end{array}\right]$$

According to the above, the establishment of SINS/DVL integrated navigation is completed.

## 3. SINS/DVL Fault Detection and Beam Failure Processing

**Figure 3:**
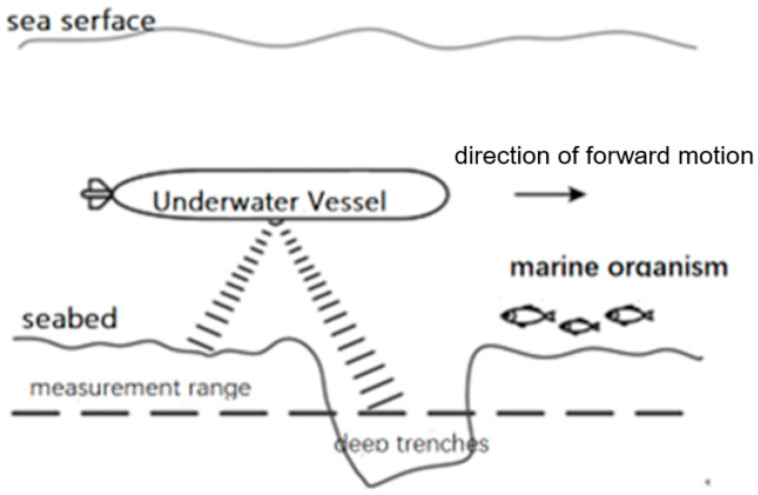
Failure of DVL velocity measurement.

In the bottom-to-bottom operation mode of the DVL, the complex underwater working environment presents challenges that can compromise the reliability of DVL beam functionality. For instance, when an Autonomous Underwater Vehicle navigates underwater, various factors such as the distance between the carrier and the seabed exceeding the DVL’s measurement range, the presence of sound-absorbing geology on the seabed, or obstruction of the beam transmission by underwater organisms can occur [21], as shown in Figure 3.

Under these conditions, it is common for two or more beams emitted by the DVL to fail to reach the seabed. Consequently, the DVL becomes unable to accurately calculate the three-dimensional velocity of the carrier or provide velocity correction information to the integrated navigation system. As a result, the integrated navigation system experiences a failure to function properly. Without access to DVL velocity values or external observation velocity to aid the SINS, the system reverts to operating solely as a pure inertial navigation system. This transition leads to the accumulation of errors over time.

Hence, in a complex underwater environment, it becomes imperative to verify the operational status of the Doppler Velocity Log (DVL) and ascertain whether it is functioning normally. Moreover, it is essential to enhance the integrated navigation filtering algorithm to accommodate the underwater noise environment, thereby improving the system’s anti-error performance [22,23].

### 3.1. Beam Fault Detection Based on Innovation

To mitigate the impact of outlier measurements on the positioning accuracy of the integrated navigation system when invalid beams are present, it is essential to preprocess the DVL velocity measurement information to detect whether the measurement values are valid. In this paper, an outlier detection method based on covariance is employed to construct a decision factor for detecting outlier measurements of DVL beams.

The difference between the Kalman filter measurement estimate and the DVL velocity measurement in the combined system is referred to as the innovation. It represents the error of the measurement estimate and is expressed by the following formula:

(36) $$\varepsilon_{k}=Z_{k}-{\hat{Z}}_{k/k-1}=H_{K}X_{K}+V_{K}-H_{K}{\hat{X}}_{k/k-1}=H_{K}{\tilde{X}}_{k/k-1}+V_{K}$$

Its variance is as follows:

(37) $$E\left[\varepsilon_{k}\varepsilon_{k}^{T}\right]=H_{K}P_{k/k-1}H_{K}^{T}+R_{K}$$

We define the decision factor s

(38) $$s=\left|\frac{tr(\varepsilon_{k}\varepsilon_{k}^{T})}{tr\left(H_{K}P_{k/k-1}H_{K}^{T}+R_{K}\right)}\right|$$

If the DVL velocity measurement data are an abnormal outlier, the innovation will mutate, and $tr(\varepsilon_{k}\varepsilon_{k}^{T})\gg tr\left(H_{K}P_{k/k-1}H_{K}^{T}+R_{K}\right)$. After detecting the abnormal outlier data output after DVL beam failure, it needs to be processed. Therefore, the decision threshold $T_{max}$ can be set according to the specific environment and performance analysis to determine whether the decision factor exceeds the set routine range.

(39) $$\left\{\begin{array}{l} \;\;s\leq T_{max},\mathrm{Normal}\;\\ \;\;s\geq T_{max},\mathrm{Abnormal}\; \end{array}\right.$$

When an abnormal value of the DVL beam is detected, the beam fault should be further judged. Therefore, in a tight coupling navigation system, according to the principle of the adaptive filtering algorithm, when one or more beams are abnormal, it is necessary to moderately increase the corresponding measurement noise characteristic $R_{K}$, and when the measurement information of the beam is an abnormal outlier, it is only necessary to set the corresponding measurement noise characteristic $R_{K}$ to infinity.

According to the simulated integrated navigation system noise environment in the simulation environment, the threshold $T_{max}$ is set to 10.

### 3.2. Improved Adaptive Filtering Algorithm

In the standard Kalman filter system, the structural parameters and noise statistical characteristics need to be known in order to achieve the optimal estimation. In practice, when the external information changes, the system noise characteristic $Q_{K}$ and the measurement noise characteristic $R_{K}$ may change, resulting in system divergence [24,25]. Using the Sage–Husa algorithm to estimate the statistical characteristics of noise in real time can restrain the divergence well.

(40) $${\hat{R}}_{k}=\left(1-d_{k}\right){\hat{R}}_{k-1}+d_{k}\left(\varepsilon_{k}\varepsilon_{k}^{T}-H_{k}P_{k/k-1}H_{k}^{T}\right)$$

(41) $${\hat{Q}}_{K}=\left(1-d_{k}\right){\hat{Q}}_{K-1}+d_{k}\left(K_{k}\varepsilon_{k}\varepsilon_{k}^{T}K_{k}^{T}+P_{k}-F_{k}P_{k-1}F_{k-1}\right)$$

(42) $$d_{k}=\left(1-b\right)/\left(1-b^{k+1}\right)$$

However, in practice, with the iteration of the filter system, the problem of a negative positive definite matrix may occur. Therefore, the value range of the decision factor s mentioned above is need to be determined, and the upper threshold $T_{max}$ and lower threshold $T_{min}$ are set. In each filtering process, s is judged and the variance of innovation is modified in real time.

(43) $$\left\{\begin{array}{c} \varepsilon_{k}\varepsilon_{k}^{T}=T_{min}\times \left(H_{K}P_{k/k-1}H_{K}^{T}+R_{K}\right),s<T_{min}\\ \varepsilon_{k}\varepsilon_{k}^{T}=T_{max}\times \left(H_{K}P_{k/k-1}H_{K}^{T}+R_{K}\right),s>T_{max} \end{array}\right.$$

When s is in the normal working range, there is no need to adjust the variance of the innovation. The problem of the negative positive definite matrix in $R_{K}$ estimator is avoided effectively when the integrated navigation system works normally and the innovation is small.

In the traditional Sage–Husa filtering algorithm, the statistical characteristics of noise need to be recalculated every time the state estimation is performed. Since $Q_{K}$ usually does not change in a stable environment, there is no need to re-estimate the $Q_{K}$ value, reducing the amount of computation. According to the measurement anomaly determination method mentioned above, when the measurement is abnormal, the statistical characteristics of the measurement noise will change greatly, and then it will be adjusted. Usually b takes a constant value in the range of 0.9~0.99, and in the actual working environment, the external conditions are constantly changing, and keeping b constant will affect the adaptive ability [26,27]. In order to make the weighting coefficient change with the change of the measurement noise, it is necessary to adjust the weighting coefficient. s as a decision factor related to innovation represents the stability of system measurement well. Therefore, the measurement noise $R_{K}$ is estimated in real time by combining the decision factor. Finally, the $R_{K}$ estimation formula in the Sage–Husa algorithm is changed as follows:

(44) $${\hat{R}}_{k}=\left(1-0.1^{\log_{{T_{max}}^{s}}}\right){\hat{R}}_{k-1}+0.1^{\log_{{T_{max}}^{s}}}\left(\varepsilon_{k}\varepsilon_{k}^{T}-H_{k}P_{k/k-1}H_{k}^{T}\right)$$

**Figure 4:**
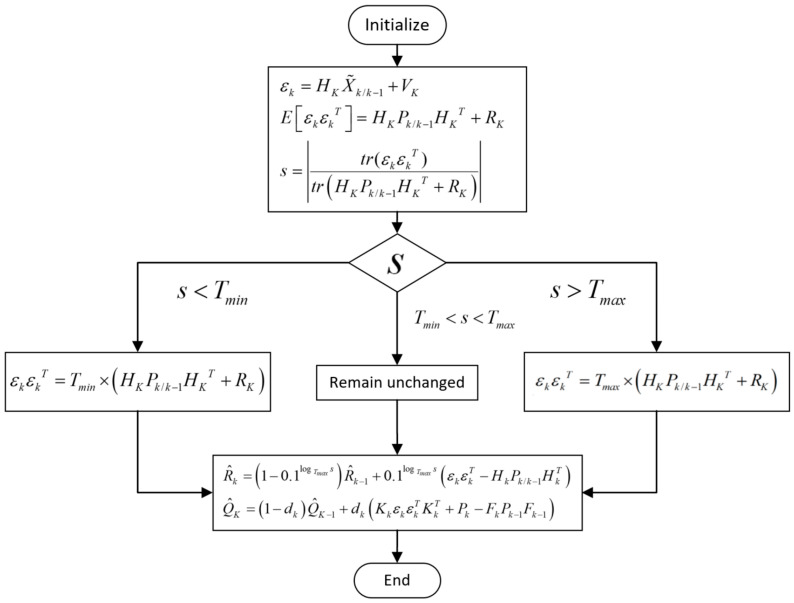
Flowchart of improved adaptive filtering algorithm.

The flowchart of the improved adaptive filtering algorithm is shown in Figure 4. The improved adaptive filtering algorithm uses the decision factor of the innovation, recalculates the covariance of the innovation when the innovation has a large fluctuation, re-estimates the measurement noise $R_{K}$ with the calculated innovation, and uses s to adjust the weighting coefficient in the estimation, which restrains the divergence of the adaptive filtering system and improves the robustness of the system.

## 4. Experiment and Result Analysis

### 4.1. Simulation Experiment

To validate the effectiveness of the proposed algorithm, a simulation experiment of the SINS/DVL integrated navigation system was conducted in the MATLAB environment to simulate Autonomous Underwater Vehicle (AUV) navigation underwater.

The sensor parameter settings in the simulation experiment are shown in Table 1.

**Figure 5:**
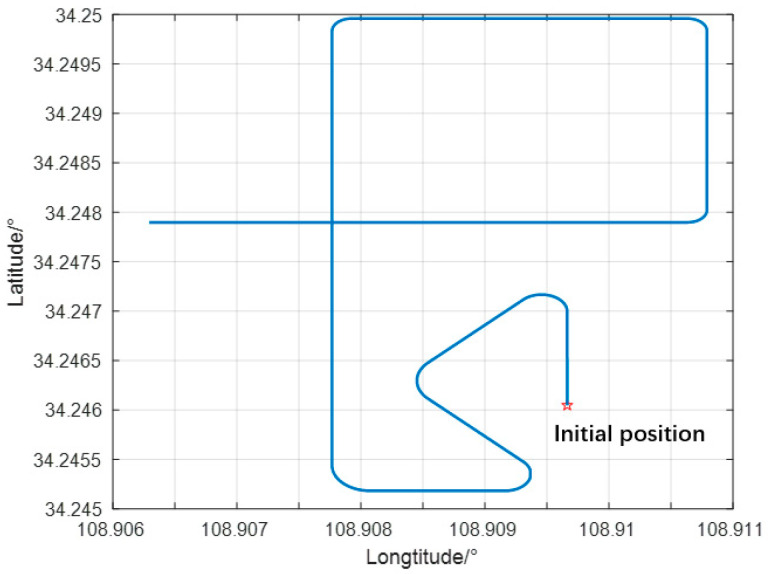
Simulation trajectory diagram.

Under identical simulation conditions, to mimic the potential miscalibrations of an AUV in a real underwater environment, ambient noise is intensified by tenfold between 500 s and 900 s to replicate the complexities of underwater conditions for a designated period, such as AUVs continuously passing large ships on the surface of the water while performing underwater operations. Subsequently, the combined navigation accuracy of the classical Kalman filter, Sage–Husa adaptive filter, and improved adaptive filter is compared under these circumstances. The simulated motion trajectory is depicted in Figure 5. Under normal working conditions, the s value ranges from 1 to 10. Therefore, in the simulation experiment, $T_{max}$ was set to 10 and $T_{min}$ to 0.8.

The simulation experiments employed the following three navigation schemes:

Navigation Scheme 1: Utilizing the conventional Kalman filter for navigation within the coupled systems.

Navigation Scheme 2: Employing the Sage–Husa filter for navigation within the coupled systems.

Navigation Scheme 3: Utilizing the improved adaptive filtering method for navigation within the coupled systems.

During the 500 s–900 s segment, severe noise leads to abnormal values of the decision factor s. This indicates the abnormal functioning of the DVL during this period, necessitating a re-estimation of the measurement noise in the filtering algorithm based on the external environment.

**Figure 6:**
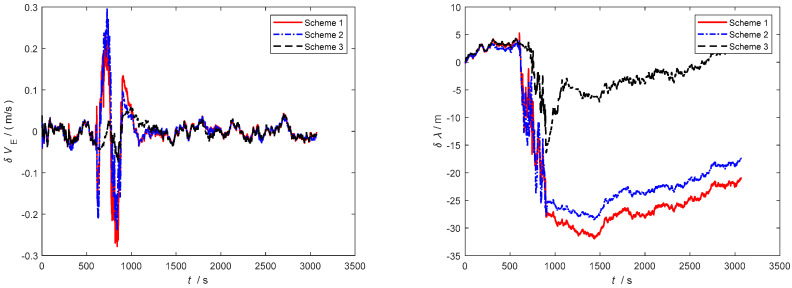
Eastbound velocity and position error.

**Figure 7:**
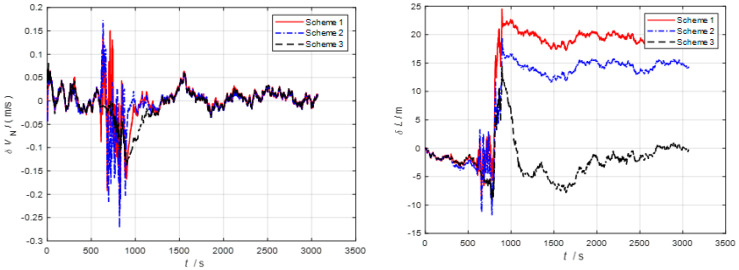
Northbound velocity and position error.

**Figure 8:**
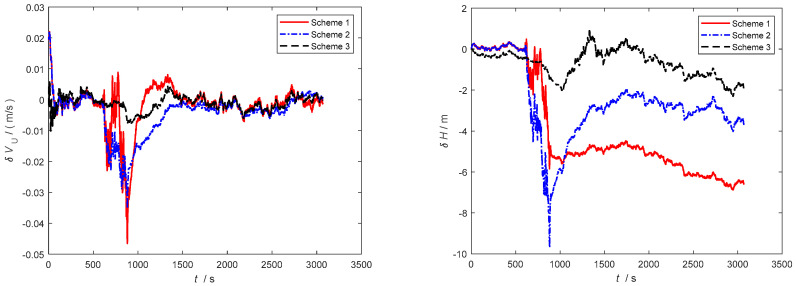
Horizontal velocity and position error.

Furthermore, as observed in Figure 6, Figure 7 and Figure 8, severe noise results in varying degrees of deviation in Scheme 1 and Scheme 2, while the speed error in the improved adaptive filtering scheme remains relatively stable. Notably, no significant deviations in position occur during the simulation.

**Figure 9:**
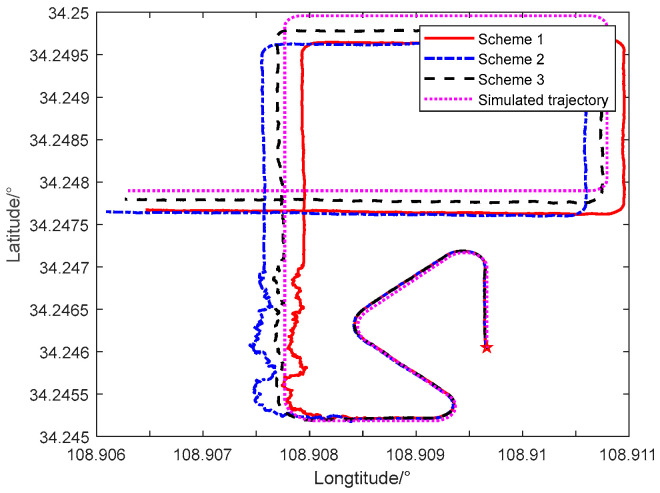
Trajectory comparison diagram of three navigation schemes.

The navigation track diagrams of the three navigation schemes are illustrated in Figure 9, revealing that the navigation track error of the improved adaptive filtering algorithm in integrated navigation is minimal.

Under identical simulation conditions, the simulation duration is extended to 3600 s. Additionally, environmental noise is intensified tenfold between 2500 s and 2900 s to simulate the underwater complex environment for a designated period. Concurrently, to simulate DVL beam failure scenarios in real underwater environments, the output speed information of the DVL beam is set to 0 every 200 s, simulating intermittent beam anomalies. Subsequently, the combined navigation accuracies of the three integrated navigation algorithms—namely, the precision Kalman filter, Sage–Husa adaptive filter, and improved adaptive filter—are, respectively, compared under these conditions.

**Figure 10:**
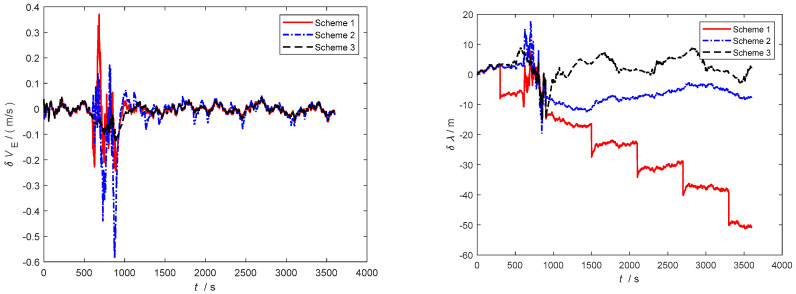
Eastbound velocity and position error in the second simulation.

**Figure 11:**
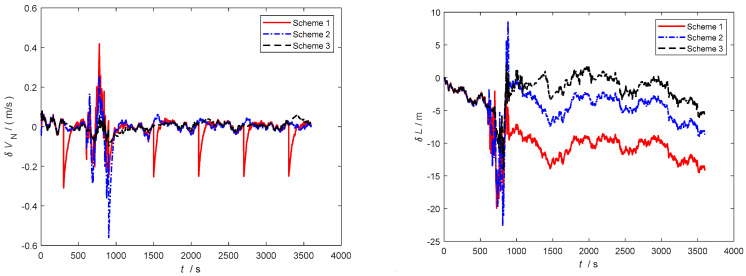
Northbound velocity and position error in the second simulation.

As depicted in Figure 10 and Figure 11, throughout the entire integrated navigation process, the speed errors of the three filtering algorithms do not exhibit significant accumulation over time. However, the improved adaptive filtering algorithm demonstrates superior stability compared to classical Kalman filtering and Sage–Husa adaptive filtering. Moreover, the improved adaptive filtering algorithm exhibits the smallest speed error in integrated navigation when compared to the other two filtering methods.

This enhanced performance of the improved adaptive filtering algorithm can be attributed to its ability to not only suppress filter divergence but also dynamically adjust the threshold value to estimate measurement noise when the decision factor is excessively high. Consequently, the influence of observation information on the current state is reduced when the error of historical observation information is substantial.

In the simulation result, it is evident that the performance of the improved adaptive filtering algorithm surpasses those of the other two methods, both in terms of the peak position error observed during the simulation process and the final position error result at the end of the simulation. Notably, the conventional Kalman filter exhibits significant deviation in position after multiple occurrences of DVL outliers. This underscores the superior accuracy and stability of the improved adaptive filtering algorithm in navigating complex underwater environments.

Therefore, in SINS/DVL integrated navigation applications, the improved adaptive filtering algorithm outperforms the classical Kalman filtering and Sage–Husa adaptive filtering algorithms in terms of stability and accuracy.

Table 2 presents the statistical results of the navigation position errors, with the relative error indicating the ratio of the maximum position error to the distance. As observed in the table, the eastern position error values for the three navigation schemes are 50.7 m, 16.9 m, and 16.6 m, respectively. Similarly, the north position error values are 20.0 m, 22.7 m, and 17.3 m, respectively. Additionally, the relative position error values of the navigation results are 52.1 m, 10.5 m, and 6.2 m, respectively.

Table 3 presents the RMS error for simulation. Comparing these results, the proposed algorithm demonstrates significant improvements in navigation position accuracy.

### 4.2. Lake Experiment

**Figure 12:**
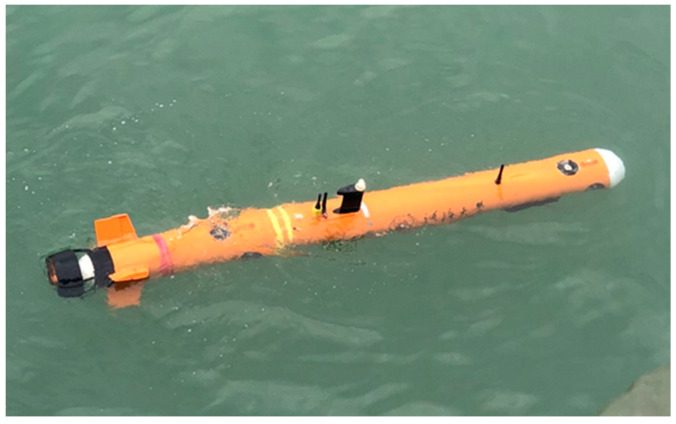
Sprite E200D miniature AUV.

To verify the overall feasibility of using various correction methods in combination, a lake test analysis of the SINS/DVL integrated navigation system was conducted. The Genie E200D small AUV served as the test platform, as illustrated in Figure 12. The primary navigation sensors in the SINS/DVL integrated positioning and navigation system include the optical fiber strapdown inertial navigation system and a four-beam Janus Doppler log. Additionally, the AUV platform is equipped with a differential GPS, a sound velocity measuring instrument, an attitude measuring instrument, a depth meter, and other auxiliary measurement modules.

The main performance indexes of the experimental equipment are shown in Table 4.

**Figure 13:**
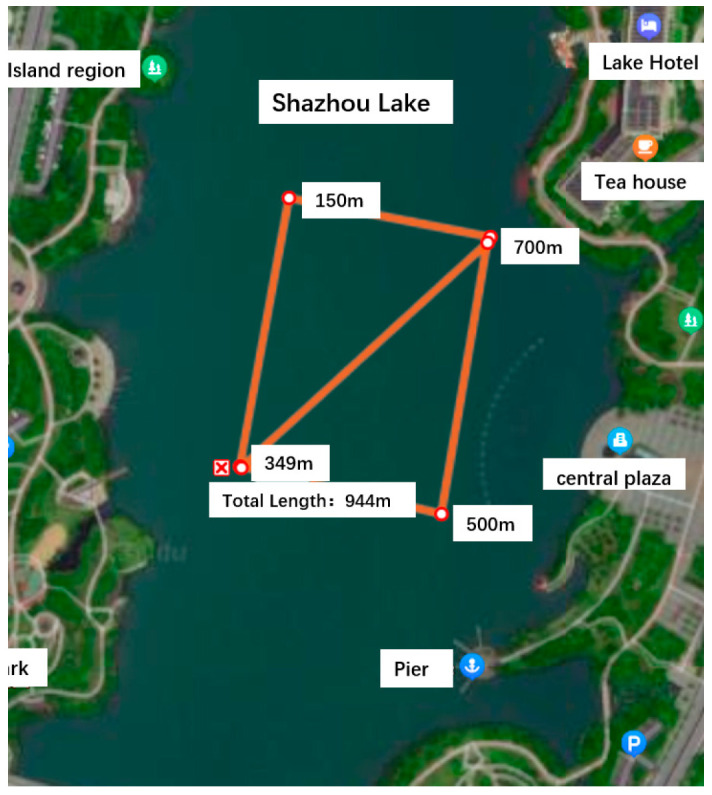
Experimental preset trajectory.

**Figure 14:**
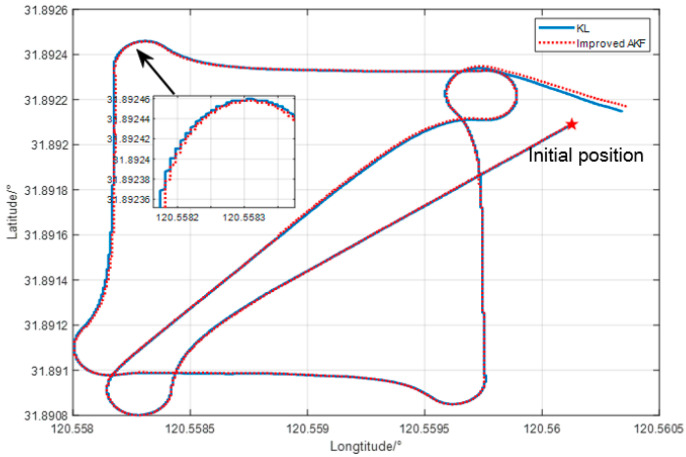
Comparison diagram of experimental trajectories.

The experimental scheme design is as follows. The AUV operates at a speed of 1.2 m/s, the path is a 150 m square side length plus diagonal line, and the sailing time is 1200 s. The experimental design route is shown in Figure 13. The traditional Kalman filter algorithm and the improved adaptive filter algorithm are used for integrated navigation experiments. As shown in Figure 14, the experimental data tracks under the two algorithms have little difference in the real water surface environment, which verifies the stability of the improved adaptive filtering algorithm in the conventional real environment.

## 5. Conclusions

This paper primarily focuses on the SINS/DVL integrated positioning and navigation system, analyzing and constructing the error models of SINS and DVL in detail, and developing the mathematical model of the SINS/DVL integrated navigation system. The study investigates data anomalies in the SINS/DVL integrated navigation system within actual underwater environments, which often result in divergence of the integrated navigation system. While the Sage–Husa adaptive filtering algorithm has historically been employed to mitigate such divergence, its filtering system faces challenges of inaccurate estimation in real integrated navigation systems, particularly in coping with the complexities of highly dynamic underwater environments.

To tackle this issue, this paper presents a novel approach centered on establishing thresholds leveraging the decision factor of innovation and subsequently adjusting the innovation’s variance. In the simulation experiment, we employ a technique involving noise amplification and the introduction of outlier values to replicate the complexities of underwater environments. Compared with the navigation results of the conventional KF filter algorithm and the improved adaptive filter algorithm, it is proved that the filter algorithm runs stably in the actual test and improves the positioning accuracy to a small extent. Comparative analysis against three methods reveals that the enhanced adaptive filtering algorithm demonstrates superior accuracy and stability compared to both the traditional Kalman filter and the Sage–Husa adaptive filtering algorithm, under conditions of noise mutation or beam failure.

Due to the limitations of the current experimental conditions, it is difficult to simulate the noise situation in a complex underwater environment. We chose to conduct experiments in lake water to verify the practicality and stability of the algorithm outside the simulation environment. Furthermore, even without setting up a complex underwater environment, the real lake water environment still presents various complexities. The experimental setup for a complex underwater environment is also a direction for further research and development that we need to pursue in the future.

## Figures and Tables

**Table 1:** Parameter settings of the sensors.

Sensors	Parameter	Value
Gyroscope	Biases drift	0.03°/h
	Random walk noise	0.01°/$\sqrt{h}$
Accelerometer	Biases drift	100 μg
	Random walk noise	50 μg
DVL	Scale factor error	0.1%

**Table 2:** Simulated position error.

	Eastbound Position Error/m	Northbound Position Error/m	Relative Position Error/m
Kalman filter	50.7	20.0	52.1
Sage–Husa filter	16.9	22.7	10.5
Improved adaptive filter	16.6	17.3	6.2

**Table 3:** RMS error for simulation.

	${\delta V}_{E}$/(m/s)	$\delta V_{N}$/(m/s)	$\delta V_{U}$/(m/s)	$\delta P_{E}$/m	$\delta P_{N}$/m	$\delta P_{U}$/m
Kalman filter	0.0682	0.0654	0.0190	22.0380	37.6062	13.4920
Sage–Husa filter	0.0393	0.0641	0.0103	18.4179	15.8249	3.7923
Improved adaptive filter	0.0181	0.0256	0.0043	11.2491	4.9841	3.7745

**Table 4:** Main performance indicators of the experimental equipment.

Laboratory Equipment	The Performance Parameters	Parameters
SINS	Accelerometer accuracy and maximum range	5 × 10^−5^ G; ±15 g
	Gyro accuracy and maximum range	0.001°/h; ±200°/s
DVL	Speed measuring precision	0.2% of 1 mm/s±
	Underground height survey	0.3~110 m
DGPS	Positioning accuracy	0.1 m
Sound speed meter	Speed measuring precision	0.1 m/s
Attitude measuring instrument	Three-axis rotary accuracy	0.001°
Depth gauge	Accuracy of measurement	±0.25% FS

## Data Availability

Dataset available on request from the authors.
